# Isolated superior mesenteric artery dissection presenting as abdominal pain in a patient with ureteral carcinoma: a case report and literature review

**DOI:** 10.3389/fsurg.2026.1863147

**Published:** 2026-08-28

**Authors:** Jia Zheng, Lei Yang, Lianrui Guo

**Affiliations:** 1 Department of Vascular Surgery, Xuanwu Hospital, Capital Medical University, Beijing, China; 2 Department of Radiology, Characteristic Medical Center of Chinese People's Armed Police Force, Tianjin, China

**Keywords:** abdominal pain, diagnosis, isolated superior mesenteric artery dissection, peritoneal metastasis, treatment

## Abstract

Isolated superior mesenteric artery dissection (ISMAD) is a rare vascular surgical disease. Its symptoms have no obvious specificity, and it is easy to be misdiagnosed or missed. This paper reports a patient with peritoneal metastasis who was eventually diagnosed with ISMAD presenting with abdominal pain, highlighting the diagnostic challenge and the importance of considering vascular emergencies in oncology patients.

## Background

Isolated superior mesenteric artery dissection is a rare vascular lesion, and its clinical manifestations can range from asymptomatic to acute intestinal ischemia. This disease usually occurs in patients without a clear history of trauma or atherosclerosis. However, among patients with malignant tumors, there are very few reports of ISMAD. We describe a patient with peritoneal metastases who was found to have ISMAD due to abdominal pain. This finding suggests that in patients with tumors, non-neoplastic vascular emergencies should still be included in the differential diagnosis to avoid delays in treatment.

## Case description

**Figure 1:**
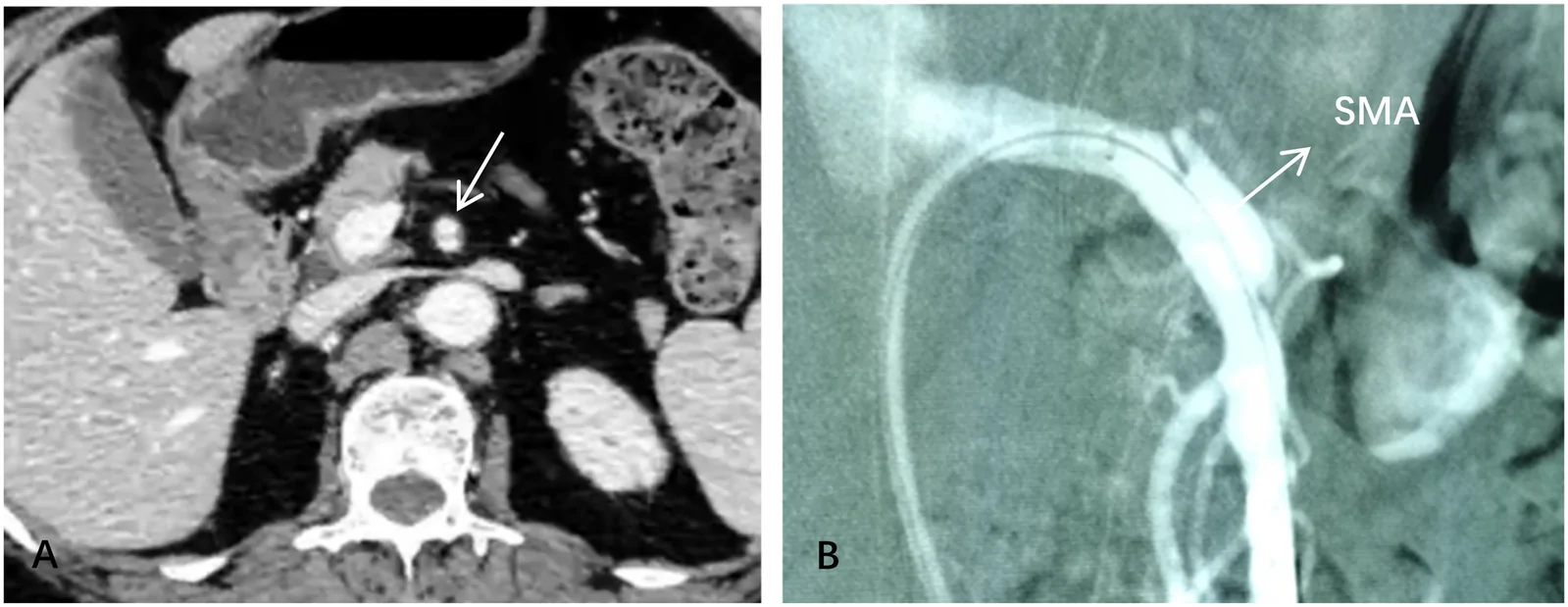
Preoperative imaging findings. **(A)** CTA showing the “double-track” sign at the proximal superior mesenteric artery (white arrow). **(B)** DSA confirming saccular dilation at the proximal segment of the SMA (white arrow).

The case we report is a 66-year-old woman who came to the hospital with abdominal pain for one week. The patient had a body mass index of 25 kg/m^2^ and no history of smoking. Laboratory tests on admission revealed hyperlipidemia. Family history was negative for vascular dissection, aneurysms, or connective tissue disorders such as Ehlers-Danlos syndrome or Marfan syndrome. Physical examination showed no signs of connective tissue disease. No significant psychosocial stressors or history of substance abuse were identified. The patient developed abdominal pain without an obvious cause a week ago, and the pain worsened after eating, accompanied by nausea and vomiting. Symptoms eased slightly after vomiting. The pain was not related to the change of body position, and there was no radiating pain in the shoulder or back. Tracing the patient's history, she had high blood pressure for years. One year prior to the current presentation, the patient was diagnosed with ureteral carcinoma at an outside hospital, where she underwent surgical resection. Detailed histopathological information, including tumor stage and grade, was not available. Peritoneal metastasis was found by PET-CT six months after surgery. We did a physical examination of the patient and found no significant positive signs other than mild tenderness in the upper abdomen. Laboratory tests such as blood routine, biochemistry, CRP, PCT, and so on are normal. We then performed a routine abdominal ultrasound and also found no abnormalities. At this point, the clinical picture—including the absence of peritoneal signs, normal laboratory findings, and unremarkable ultrasound—did not suggest an acute surgical abdomen. Given the patient's known peritoneal metastasis, the abdominal pain was initially attributed to malignancy-related causes, a common clinical scenario in patients with advanced cancer. We therefore initiated symptomatic therapy, including fluid resuscitation, acid suppression, antiemetics, and antispasmodics. However, the patient's symptoms persisted despite this treatment. The postprandial pattern of pain and the lack of alternative explanations prompted us to reconsider the initial diagnosis. A multidisciplinary consultation was convened, and computed tomography angiography (CTA) was performed to exclude vascular pathologies. CTA revealed proximal septate changes in the superior mesenteric artery (SMA) and poor distal filling. Subsequent digital subtraction angiography (DSA) confirmed a local bulge at the proximal end of the SMA with sac-like dilation, measuring 18.5 mm in length with a false-to-true lumen ratio of 1.2. Based on the Sakamoto classification, the lesion was categorized as type I (Figure 1). This decision proved critical in establishing the correct diagnosis.

**Figure 2:**
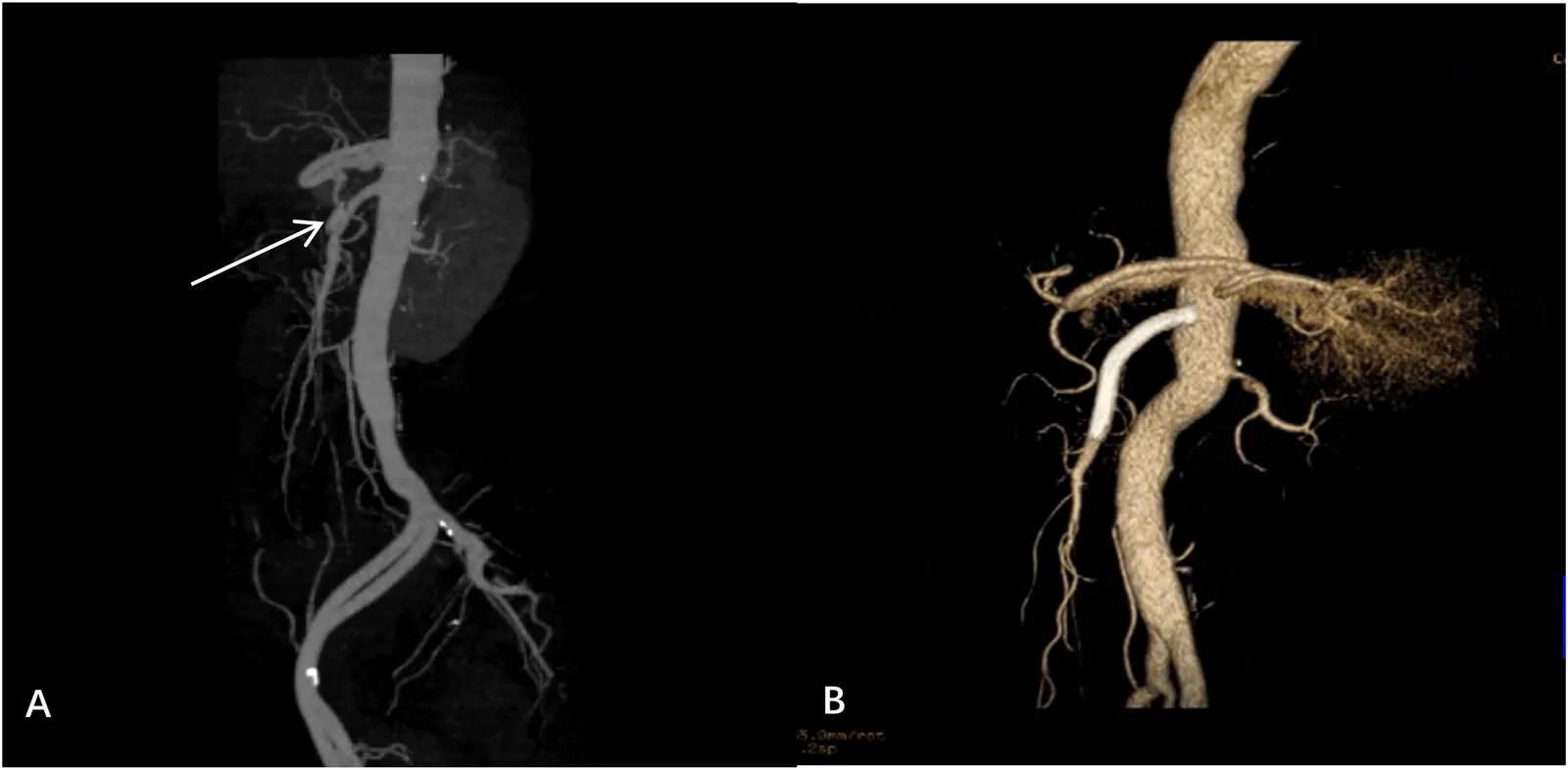
Three-dimensional CTA reconstruction. **(A)** Preoperative imaging showing the dissected segment (white arrow). **(B)** One-week postoperative imaging demonstrating stent patency and unobstructed blood flow.

During the operation, a 5 mm in diameter×5 cm in length covered stent (Gore Viabahn) was deployed into the superior mesenteric artery. Intraoperative imaging examination confirmed resolution of the dissection and satisfactory distal flow. Following the procedure, the patient received dual antiplatelet therapy (aspirin 100 mg daily and clopidogrel 75 mg daily) for three months, followed by aspirin monotherapy. Blood pressure was strictly controlled with antihypertensive agents. One week after the operation, the patient underwent the first reexamination. The imaging examination indicated a good recovery (Figure 2). Six months after the procedure, the patient was followed up by telephone. She reported no recurrence of abdominal pain, no anorexia, and no weight loss. However, imaging reexamination at 6 months was not performed because the patient was asymptomatic and declined further hospital visits. This represents a limitation of our follow-up assessment, which is acknowledged in the Discussion section. The clinical timeline is summarized in Table 1.

**Table 1:** Clinical timeline of the patient.

Time	Event
1 year prior	Diagnosed with ureteral carcinoma; underwent surgical resection
6 months prior	PET-CT revealed peritoneal metastasis
1 week prior	Onset of postprandial abdominal pain, nausea, vomiting
Day 1 of admission	Physical examination (mild upper abdominal tenderness); laboratory tests (normal); abdominal ultrasound (unremarkable)
Day 3 of admission	Symptomatic therapy failed to improve symptoms; persistent postprandial pain prompted vascular imaging consideration
Day 4 of admission	Multidisciplinary consultation; CTA revealed SMA dissection
Day 5 of admission	DSA confirmed diagnosis; endovascular stenting performed
Postoperative day 1	Abdominal pain significantly relieved
Postoperative day 7	CTA reexamination showed good stent patency and unobstructed branch flow

## Patient perspective

The patient shared her experience as follows: “When the abdominal pain started, I thought it was related to my cancer. When the doctors told me it was actually a problem with my blood vessel, I felt a sense of relief—not because it wasn't serious, but because it was treatable and not a progression of my cancer. After the procedure, the pain disappeared immediately. I was able to eat normally again, which I hadn't been able to do for weeks. Now, six months later, I feel well and have returned to my daily activities. I am truly grateful that the doctors did not simply assume it was my cancer and continued to investigate other possible causes.”

## Discussion

Isolated superior mesenteric artery dissection is a rare clinical disease, accounting for only 6.9% of visceral artery dissection (1). The disease was first reported by Bauersfeld in 1947 and was diagnosed by autopsy in the early years. Nowadays, due to the progress of CT technology, its detection rate is gradually increasing. In patients with ISMAD, the prevalence is significantly higher in men than in women, with a ratio of approximately 3:1 (2). Older people aged 50–70 years are more likely to develop the disease (3), which may be related to vascular degenerative changes. Currently, most of the reported cases have been concentrated in Asia, especially in East Asian countries (4).

The etiology of ISMAD has not been fully identified, but it is regarded as a complex interplay of various factors, including genetic predispositions and underlying vascular conditions. From an anatomical point of view, the superior mesenteric artery has two characteristic segments: a proximal fixed segment (located behind the pancreas) and a distal free segment (running along the mesenteric root), which form physiologically curved segments between them (5). The arterial wall in this area is most affected by shear stress and has significant hemodynamic abnormalities, which is a high incidence site of dissection (6). After dissecting the superior mesenteric artery, Kang et al (7) believed that systemic vascular inflammations such as giant cell arteritis and Takayasu arteritis might be related to this disease. In this case, the absence of family history and clinical signs of connective tissue disease made an inherited predisposition unlikely. The patient had no smoking history, and hyperlipidemia was the only identifiable risk factor, although its direct contribution to ISMAD remains uncertain.

Although the exact etiology of ISMAD remains unclear, several factors have been implicated, including genetic predispositions such as Ehlers-Danlos syndrome and familial thoracic aortic dissection (8, 9), as well as conditions like fibromuscular dysplasia that may compromise arterial wall integrity (10).

There have been previous reports indicating that patients with colon cancer and stomach cancer also have an increased risk of developing ISMAD (11). Researchers believe that this can be explained by systemic tumor inflammation and anatomical disorders caused by space-occupying effects. However, the discovery of ISMAD in ureteral malignant tumors has been reported for the first time.

In our patient, the co-occurrence of ureteral carcinoma and ISMAD raises the question of whether a causal or predisposing relationship exists. Although a direct causal link cannot be definitively established, several possible mechanisms warrant consideration. First, malignancy-related hypercoagulability may contribute to vascular injury and dissection. Second, systemic inflammation associated with tumor burden may promote arterial wall remodeling and weaken vascular integrity. Third, although the patient did not receive chemotherapy or radiotherapy prior to the dissection, such treatments have been reported to cause vascular toxicity in other settings. More importantly, regardless of whether a direct causal relationship exists, this case provides a valuable clinical lesson: in cancer patients with new-onset abdominal pain, vascular emergencies should remain in the differential diagnosis, and CTA should be considered when the clinical presentation is atypical.

The symptoms of superior mesenteric artery dissection do not have obvious specificity and are rather difficult to distinguish. Approximately 55% of patients present with acute or chronic abdominal pain as the initial symptom, often accompanied by radiating pain in the back (4.8%) (12). It is worth noting that patients often experience more severe pain after meals. This might be due to an increased demand for blood flow in the gastrointestinal tract, but the arteries become ischemic due to dissection. Auxiliary examinations play a crucial role in the diagnosis of ISMAD. Computed tomography (CT) is commonly employed, with studies indicating that features such as the “double-track sign” are characteristic of the disease (13). Furthermore, the perivascular fat surrounding the diseased blood vessels may also be helpful for the diagnosis of ISMAD (14).

The particularity of our case in this report is that there was a misdiagnosis in determining the cause of the patient's pain due to the presence of peritoneal metastases. It is well known that abdominal pain is a common symptom of peritoneal metastases (15). Therefore, when the patient developed symptoms and after ruling out an acute abdomen, we inferred that the abdominal pain might be related to peritoneal metastasis. However, the final result reflects our mistake in judging the cause of the disease. Generally speaking, abdominal pain caused by peritoneal metastasis tends to be slow in onset and insensitive. The location of the pain depends on the position of the primary lesion. The pain caused by peritoneal metastasis of ureteral cancer is often located in the lower abdomen or waist. For ISMAD, the typical feature is pain after eating, and changes in body position may aggravate or relieve the pain. Overall, the most significant identification method is still computed tomography angiography (CTA). When patients present with characteristic abdominal pain after eating and have hypertension, ISMAD should be highly suspected. CTA examination is helpful for our diagnosis of this disease. Similarly, other uncommon vascular emergencies may present with atypical abdominal symptoms and mimic more common gastrointestinal conditions, as highlighted by a recent case report of spontaneous splenic rupture initially misdiagnosed as peptic ulcer disease (16). This underscores the importance of maintaining a broad differential diagnosis for abdominal pain, particularly when the clinical course is atypical.

In addition, the differential diagnosis of abdominal pain in elderly patients is broad. In this case, the initial diagnostic consideration was peritoneal metastasis-related pain, which is typically characterized by chronic dull pain with variable location. However, other common etiologies were systematically considered. The absence of elevated amylase/lipase and normal liver function tests largely excluded acute pancreatitis and biliary pathology. The normal abdominal ultrasound and lack of peritoneal signs argued against acute cholecystitis or perforated viscus. Peptic ulcer disease was considered less likely given the absence of risk factors and the lack of response to acid suppression therapy. The postprandial pattern of pain, its severity disproportionate to physical findings, and the failure of symptomatic therapy prompted further vascular imaging. CTA ultimately confirmed the diagnosis of ISMAD. This case illustrates that in cancer patients with atypical abdominal pain, a broad differential diagnosis—including rare vascular emergencies—should be maintained, and CTA should be considered when the clinical course is unexplained.

The treatment of ISMAD has garnered attention due to its rarity and potential for severe complications, such as acute mesenteric ischemia. Various treatment methods have been explored, ranging from conservative treatment to surgical and endovascular interventions. The choice of treatment may depend on individual patient characteristics and clinical presentation (17). For patients with no symptoms or mild symptoms, conservative treatments such as blood pressure control and antiplatelet therapy may have better effects (18). However, for patients who receive conservative treatment, regular follow-up of CTA is necessary (19). On the other hand, for patients with severe symptoms and a dissection greater than 50 mm, stenting provides better short-term outcomes, and there are fewer complications. However, the long-term patency rate requires further study (20). Open surgery is more suitable for patients with intestinal necrosis or arterial rupture. After early diagnosis and timely intervention, the prognosis of ISMAD is still very good, and a finding that is consistent with the study by Xiong et al (21).

In this case, the decision to proceed with endovascular stenting rather than conservative management was made based on the following considerations: (1) the patient's symptoms were severe and persistent, with postprandial pain refractory to symptomatic therapy; (2) DSA revealed a significant saccular dilation at the proximal SMA, indicating a high risk of progression or rupture; (3) the dissection morphology was unfavorable for conservative treatment. After multidisciplinary discussion, endovascular intervention was performed. The choice of dual antiplatelet therapy (aspirin 100 mg daily plus clopidogrel 75 mg daily) rather than anticoagulation was based on the current standard of care for endovascular stenting of visceral artery dissections, which prioritizes prevention of in-stent thrombosis. While the patient's malignancy-related hypercoagulability was considered, anticoagulation was not routinely indicated in the absence of concurrent atrial fibrillation, venous thromboembolism, or other compelling indications. Furthermore, the covered stent used (Gore Viabahn) is associated with a low risk of acute thrombosis, and the addition of anticoagulation would have increased bleeding risk without proven additional benefit in this setting. This approach is consistent with previous reports on endovascular management of ISMAD, in which antiplatelet therapy rather than anticoagulation is typically employed for stent patency maintenance (13, 22).

## Limitations

Several limitations of this case report should be acknowledged. First, as a single-case report, our findings cannot be generalized to all patients with ureteral carcinoma or peritoneal metastasis. Second, the causal relationship between ureteral carcinoma and ISMAD remains speculative, and no genetic or connective tissue disorder work-up was performed. Third, the follow-up duration was relatively short, and the 6-month assessment relied solely on telephone interview without objective imaging confirmation due to patient refusal. Fourth, the patient had not received chemotherapy or radiotherapy, limiting our ability to comment on treatment-related vascular toxicity. Finally, pathological examination of the dissected arterial wall was not available, as no surgical resection was performed. Future studies with larger cohorts and longer follow-up are needed to better understand the association between malignancies and ISMAD.

## Conclusion

Isolated superior mesenteric artery dissection, as a rare disease, can be diagnosed through the typical manifestations of abdominal pain after eating and CTA. The case we reported gives clinicians a warning that attention needs to be paid to non-neoplastic vascular emergencies, especially ISMAD. And through individualized treatment and regular follow-up, the disease has a good prognosis.

## Data Availability

The datasets presented in this article are not readily available. Requests to access the datasets should be directed to lruiguo@sina.com.
